# In-vivo application of low frequency alternating currents on porcine cervical vagus nerve evokes reversible nerve conduction block

**DOI:** 10.1186/s42234-021-00072-w

**Published:** 2021-06-30

**Authors:** Maria Ivette Muzquiz, Lindsay Richardson, Christian Vetter, Macallister Smolik, Awadh Alhawwash, Adam Goodwill, Rizwan Bashirullah, Michael Carr, Ken Yoshida

**Affiliations:** 1 Department of Biomedical Engineering, Indiana University - Purdue University Indianapolis, Indianapolis, USA; 2 Department of Biology, Indiana University - Purdue University Indianapolis, Indianapolis, USA; 3 Weldon School of Biomedical Engineering, Purdue University, West Lafayette, USA; 4 Biomedical Technology Department, King Saud University, Riyadh, Saudi Arabia; 5 Department of Anatomy, Cell Biology, and Physiology, Indiana University School of Medicine, Indianapolis, USA; 6 Galvani Bioelectronics, Glaxo Smith Kline, GSK, King of Prussia, USA

**Keywords:** Neuromodulation, Conduction block, Nerve block, Low frequency alternating current block

## Abstract

**Background:**

This paper describes a method to reversibly block nerve conduction through direct application of a 1 Hz sinusoidal current waveform delivered through a bipolar nerve cuff electrode. This low frequency alternating current (LFAC) waveform was previously shown to reversibly block the effects of vagal pulse stimulation evoked bradycardia in-vivo in the anaesthetised rat model (Mintch et al. 2019). The present work measured the effectiveness of LFAC block on larger caliber myelinated vagal afferent fibers in human sized nerve bundles projecting to changes in breathing rate mediated by the Hering-Breuer (HB) reflex in anaesthetized domestic swine (n=5).

**Methods:**

Two bipolar cuff electrodes were implanted unilaterally to the left cervical vagus nerve, which was crushed caudal to the electrodes to eliminate cardiac effects. A tripolar recording cuff electrode was placed rostral to the bipolar stimulating electrodes on the same nerve to measure changes in the compound nerve action potentials (CNAP) elicited by the vagal pulse stimulation and conditioned by the LFAC waveform. Standard pulse stimulation was applied at a sufficient level to induce a reduction in breathing rate through the HB reflex. If unblocked, the HB reflex would cause breathing to slow down and potentially halt completely. Block was quantified by the ability of LFAC to reduce the effect of the HB reflex by monitoring the respiration rate during LFAC alone, LFAC and vagal stimulation, and vagal stimulation alone.

**Results:**

LFAC achieved 87.2 ±8.8% block (n=5) at current levels of 1.1 ±0.3 mA_p_ (current to peak), which was well within the water window of the working electrode. CNAP showed changes that directly correlated to the effectiveness of LFAC block, which manifested itself as the slowing and amplitude reduction of components of the CNAP.

**Conclusion:**

These novel findings suggest that LFAC is a potential alternative or complementary method to other electrical blocking techniques in clinical applications.

## Background

Electrical nerve conduction block provides a means to interrupt or subtractively modulate the neural activity within somatic or autonomic nerves. It has been shown to reduce spasticity via motor nerve block and modulate the activity of autonomic nerves (Kilgore and Bhadra 2014; Bhadra et al. 2006; Johannessen et al. 2017). Current techniques being investigated that have provided evidence of nerve conduction block include: kilohertz frequency alternating current block (kHFACb) (Kilgore and Bhadra 2014; Bhadra and Kilgore 2005; Bhadra et al. 2018; Patel and Butera 2015), direct current (DC) block (Whitwam and Kidd 1975; Bhadra and Kilgore 2004; Trenchard and Widdicombe 1973), anodal block (Rijkhoff et al. 1994; Thoren et al. 1977), and quasi-trapezoidal stimulation (Tosato et al. 2007; Fang and Mortimer 1991a; Fang and Mortimer 1991b; Fang and Mortimer 1991c). However, in many of these methods, side effects and shortcomings need to be addressed. DC block, for example, can result in toxic byproducts when the electrode potentials exceed the Water window resulting in hydrolysis and formation of hydronium cations and hydroxide anions. This has shown to not only irreparably injure tissues (Whitwam and Kidd 1975), but also causes electrode corrosion over time. The use of an alternating charge balanced current has the ability to reverse the Faradaic reactions in order to reduce the possibility of damaging byproduct formation. KHFACb is a method that uses a sinusoidal waveform with frequencies ranging from 1 kHz - 40 kHz (Kilgore and Bhadra 2014; Kilgore and Bhadra 2004). However, it has an associated onset response which causes the activation of nerve fibers before block can occur (Foldes et al. 2009), implicating an open state Na^+^ channel inactivation mechanism. A combination of DC block followed by kHFACb is a strategy used in which the DC waveform blocks the onset activation of kHFACb (Franke et al. 2014; Miles et al. 2007; Ackermann et al. 2011; Vrabec 2016).

During our investigation of kHFACb, we discovered that reducing the frequency of the waveform (<10 Hz) achieved phasic blocking of action potentials at low current levels in in-vivo testing on earthworm nerves without an onset response. Furthermore, the waveform was tested on ex-vivo canine and porcine vagus nerves and resulted in successful nerve conduction block. The applied waveform was a simple sinusoid characterized by amplitude and frequency, at a much lower frequency than those used for kHFACb, and thus was named Low Frequency Alternating Current block (LFACb). The LFACb phenomenon was conserved from invertebrates to vertebrates (clitellata — mammalia) and between species (canus — sus) (Horn et al. 2019). Block was achieved at current levels that are approximately half of those required for kHFACb within the linear region of the working electrode. LFACb is presented as an isolated current/voltage source via a bipolar electrode to the nerve. The currents are balanced during one cycle of the waveform reversing the chemo-electric reactions taking place on each pole at each phase. Thus, LFACb has the low threshold characteristics associated with DC block and charge balanced reversibility of kHFACb.

A companion paper describes the work with LFACb applied to nerve fibers in the cervical vagus of rats against vagal pulse stimulation activated bradycardia. Although LFACb in the rat cervical vagus was effective, size of the nerve raised the question of whether LFACb can be applied to larger “human sized” nerve bundles. The present work aims to address this question.

## Methods

### Animal and surgical prep

All experiments involving animals were approved by an Institutional Animal Care and Use Committee (IACUC) and performed in accordance with the Guide for the Care and Use of Laboratory Animals (National Institutes of Health Publication. No. 85-23, Revised 2011). Adult (∼50 kg) male domestic swine were sedated with an intramuscular injection of with telazol, xylazine, and ketamine (5.0, 2.5, and 2.5 mg/kg respectively). Swine were then intubated, placed in supine position, and anesthesia maintained via inhaled isoflurane anesthesia (0.5 - 3%). A unilateral femoral cutdown was performed and a femorally inserted catheter advanced into the thoracic aorta for continuous measurement of blood pressure (BP). BP, electrocardiograph (ECG), O_2_ saturation, respiration, and body temperature were continuously monitored through the course of the procedure. Respiration was measured through a pressure transducer attached to a respiration monitor belt (RMB-BTA, Vernier, Beaverton OR) placed around the chest of the animal. A custom software for MATLAB (2016a, Mathworks, Natick, MA) allowed for the visualization of the voltage equivalent respiration throughout the experiment. Subsequent to placement of all monitoring instrumentation, a midline anterior incision was made in the neck and the vagus nerve was bilaterally isolated. All animals breathed voluntarily and did not require forced ventilation. Upon reaching the end of the experiment the animal was euthanized under deep anaesthesia by electrical stoppage of the heart via direct DC stimulation of the heart. Pneumothorax was performed bilaterally and the heart was removed to assure death.

### Electrode configuration

**Fig. 1:**
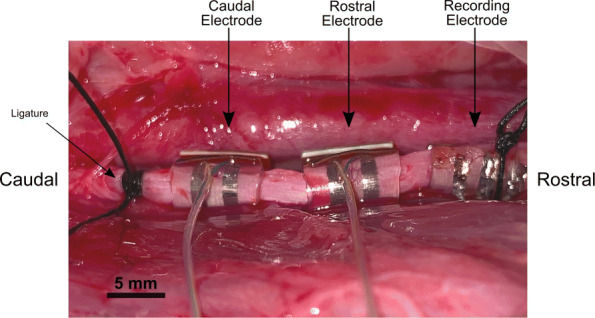
Electrode placement on the isolated, left cervical vagus of a swine for the LFAC experiments. The CE, a bipolar cuff, was used for vagal stimulation. The LFAC conditioning waveform was presented at the RE. A tripolar recording electrode for ENG recordings was placed most rostral on the vagus nerve and secured with suture. A ligature was placed caudal to all three electrodes to eliminate cardiac effects due to stimulation of the left vagus nerve

Two bipolar extrafascicular cuff electrodes were positioned on the exposed left cervical vagus shown in Fig. 1. The LFAC waveform was delivered through the rostral electrode (RE), a Platinum-Iridium bipolar cuff (2.0 mm inner diameter, ∼6.0 mm inner circumferential contact length, 1.5 mm contact width, 2.0 mm contact to end distance, 1.0 mm contact pitch; CorTec GmbH, Neuer Messplatz 3, 79108 Freiburg Germany) coated with poly-ethylenedioxythiophene (PEDOT)-based Amplicoat^®^ (Heraeus Medical Components, Saint Paul MN) (Boehler et al. 2019; Heraeus Medical Components). A second bipolar CorTec cuff was placed caudal to the RE and was used for vagal stimulation, as the caudal electrode (CE). Additionally, a custom built 3D printed 3.0 mm diameter tripolar cuff (Richardson et al. 2018) was placed most rostral on the nerve for recording compound nerve action potentials (CNAPs) and secured with a suture. The left cervical vagus was crushed caudally to all electrodes using a pair of forceps and suture to eliminate caudally directed responses due to electrical stimulation. The right vagus nerve was kept intact to maintain the stability of the preparation. Electroneurograph (ENG), LFAC waveform, respiration, and ECG signals were acquired simultaneously at 48 kHz using a Zoom FN8 sampling front end to Tracktion T7 Digital Audio Workstation (DAW). Channels whose signals had bandwidths <20 Hz, respiration and LFAC waveform, were frequency modulated using a Vetter FM Recording Adapter (model 2D) prior to acquisition via the Zoom sampling front end to Tracktion. ENG was highpass filtered at 10 Hz (2500x - 5000x gain) using a multi-channel gain-filter main amp (CyberAmp 320, Axon Instruments).

### Nerve stimulation and experimental paradigm

**Fig. 2:**
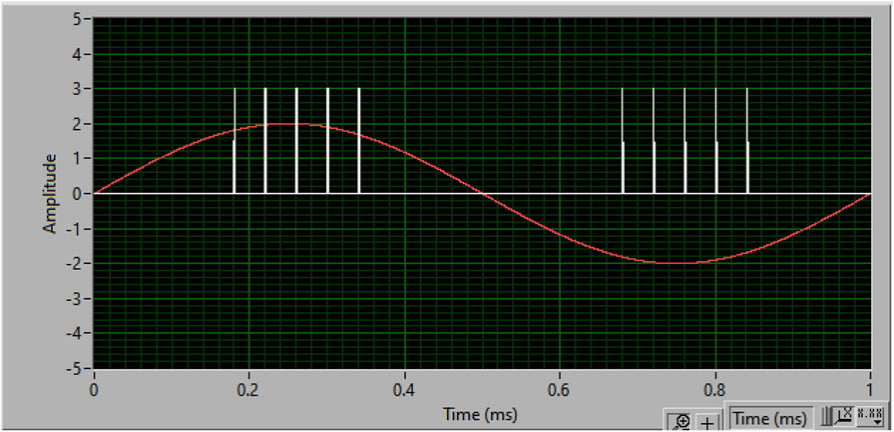
LFAC waveform at 1 Hz and sync pulses generated. The sync pulses were phased to trigger at the peaks of the sinusoidal waveform

The LFAC waveform and stimulation sync pulses were generated using an arbitrary function generator (Analog Discovery 2, Digilent Inc, Pullman WA) controlled via a custom written waveform generation routine written in LabVIEW^®^. The vagal stimulation pulse train generated by the custom written routine consisted of a train of 5 sync pulses (25 Hz pulse frequency, 200 ms train duration, and ∼170 ms train delay) delivered at 2 Hz and phased to coincide with the positive and negative peaks of the LFAC sinusoid. Prior ex-vivo and in-silico experiments (Horn et al. 2019; Yoshida and Horn 2020) showed a phasic blocking phenomena against continuous pulse stimulation. The sync pulses were applied to the vagus nerve using an opto-isolated stimulator (DS3, Digitimer LTD, Hertfordshire UK) triggered by the stimulation sync pulses. The pulse amplitude and duration were kept constant during the experimental sequence and set at the DS3 stimulator. Pulses delivered ranged between 100 - 1000 *μ*s pulse width (PW), and 0.2 and 1.2 mA pulse amplitude (PA). Figure 2 shows an example of the sync pulses and LFAC waveform generated.

Adequate stimuli were determined by visually examining the voltage equivalent respiration output on MATLAB. This was a qualitative measure. Stimulation was increased until an increase in inter breath interval was identified, indicative of the activation of the Hering-Breuer (HB) reflex. However, large ampulitude stimulation resulted in the complete cessation in breathing. Therefore, the stimulus was titrated to result in a visible effect without complete cessation of breathing.

The LFAC waveform was presented to the blocking electrode through a custom built analog optical isolator to an isolated voltage controlled current source (CS580, SRS, Sunnyvale CA or custom built current source). The optical isolations used electrically isolated the LFAC current pathways from the pulse stimulation pathways to eliminate cross-talk currents and reduce stimulation noise pickup from the electroneurogram recordings. The amplitude of the LFAC waveform was gradually increased and the voltage across the blocking electrode was monitored to remain within the Water window. The voltage across the blocking electrode was measured and acquired directly using the calibrated monitored output of the current source. The typical Water window range of the PEDOT coated electrodes is -0.9 to 0.6 V versus Ag/AgCl (Cogan 2008) for half cell potentials. Simultaneously, without reaching or exceeding the Water window, the blocking threshold of the HB reflex was recorded.

To test the effect of the LFAC waveform, the vagal stimulus train and the LFAC waveform were presented in a regular continuous sequence as follows: 1 — Pre Phase) ∼20s baseline period of no stimulation, 2 — LFAC Only Phase) ∼20s LFAC delivered to the RE, 3 — LFAC+Stim Phase) ∼20s LFAC delivered to the RE and vagal stimulation at the CE, 4 — Stim Only) ∼5 - 10s Vagal stimulation at CE, 5 — Post Phase) No stimulation return to baseline. This outlined test sequence was repeated on an average of 5.6 times in each animal to determine the effective blocking threshold. The vagal stimulation phase was maintained at minimum to avoid complete cessation in breathing. Following the testing sequences, control cases were carried in which vagal stimulation was presented at the RE and LFAC was delivered at the CE. However, due to different electrode impedance of the RE and CE, the amplitude of the block waveform had to be reduced.

### Data analysis

The analysis of the acquired data was performed using custom software written in MATLAB (2016a, Mathworks, Natick, MA). The modulated channels were demodulated using a standard FM demodulation algorithm. The peaks of each breath were located and used to calculate the instantaneous breathing rate (BRrate) and median BRrate during each of the 5 epochs outlined above. The breathing rates and ’Pre’ epoch normalized breathing rates were calculated, collected and plotted across all the test cases as raster plots. Each raster was aligned to the beginning of the ’Stim Only’ epoch. This aligment shows the difference between the case ’LFAC+Stim’ versus ’Stim Only’ case, which represents the case where the effects of vagal stimulation are not blocked. Since the resting breathing rate was different for different animals and experimental runs, the instantaneous breathing rate values were normalized with respect to the average breathing rate during each ‘Pre’ epoch. This corresponds to the breathing rate without vagal stimulation or the test case representing 100% block of the effects of vagal pulse stimulation and enabled comparison of the ’LFAC only’ and ’LFAC+Stim’ cases against the baseline breathing rate.

To calculate the percent block, the normalized *BRrate*_*N*_ during each epoch of the experimental sequence was calculated using the following equation:

1 $$ \begin{array}{c} {\kern-17.5pt}\ BRrate(\%)_{N} = \left(\!\!1\! -\! \frac{\![cond]- median\left(BRrate_{{pre}}\!\right)}{median\left(\!BRrate_{{pre}}\!\right)-median\left(\!BRrate_{{stimonly}}\!\right)}\!\right)\!*100 \end{array}  $$

Where [cond] represents the median breathing rate during each of the 5 epochs. The denominator term, (median(BRrate _*pre*_) — median(BRrate _*stimonly*_)), represents the maximum depression in the breathing rate. Quantitatively, this equation results in 100% normalized breathing rate during the ‘Pre’ epoch and 0% during the ‘Stim Only’ epoch. Thus, taking the normalized breathing rate as our measure, it correlates 1:1 to the percent block in the ‘LFAC+Stim’and ‘Stim Only’ epochs in the test and control experimental cases.

To isolate the CNAPs, the recordings made from the tripolar recording electrode were vagal nerve stimulus pulse trigger averaged in the ’LFAC+Stim’ and ’Stim Only’ cases for the test and RE/CE reversed control cases. The stimulus triggered sweeps were superimposed and averaged within the window of interest. The CNAPs are clearly visible, however, the artefact related amplifier recovery following the stimulus pulse was not removed. Attempts to eliminate the stimulation artifact with post-hoc signal processing introduced additional peaks that themselves may be artefactual. Thus, the decision was made to present the data without additional processing aside from the stimulus triggered averaging.

## Results

**Fig. 3:**
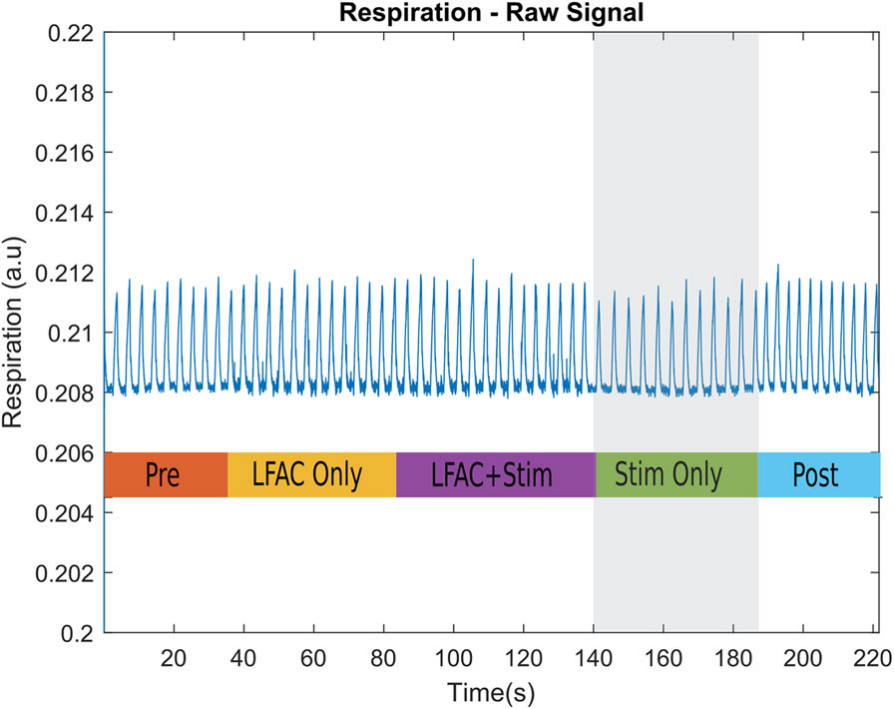
Example of a raw respiration data set during experiment. Vagal stimulation alone occurred at approximately 140 seconds, shown as the shaded region. In this period, there is an evident increase in inter-breath interval and a slight decrease in amplitude. This indicates that the HB reflex was adequately activated

Figure 3 shows an example of a continuous recording of the respiration measured in an experimental run. Superimposed on the figure are the periods indicating the different stimulation performed during the run. Upon examination, there is a clear increase in the inter breath interval at ∼140 s showing the reduced breathing rate induced by VNS mediated HB reflex. Following the ’Stim Only’ period, there is an increase in the breathing rate in the ’Post’ period.

**Fig. 4:**
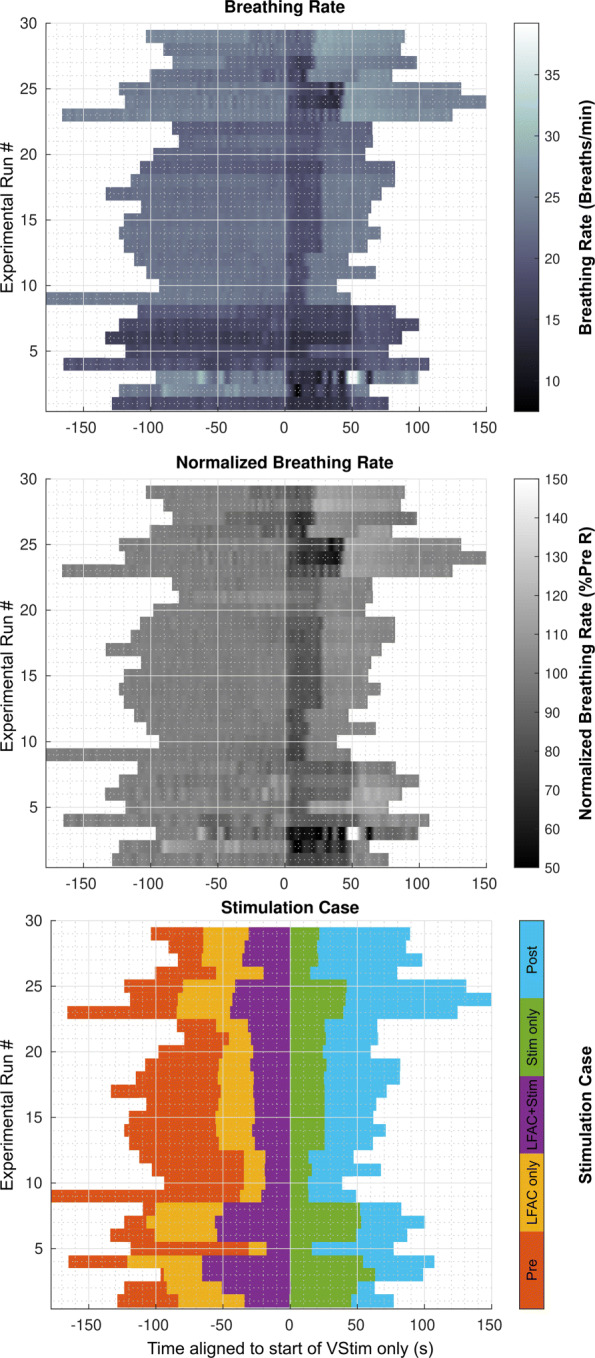
Temporal raster plots of the continuous breathing rate (Top), and normalized breathing rate (Middle) records as a function of stimulation case (Bottom) for all experimental runs across all animals (N=30). Times were aligned to the start of the ’Stim Only’ epoch to show the transition between the breathing rate during LFAC + vagal stim and vagal stimulation only, which shows a drop in breathing rate when LFAC is removed. Top panel’s breathing rate is the instantaneous rate calculated using the instantaneous interval between inspirations. The middle panel shows the instantaneous breathing rate normalized to the average ’Pre’ epoch breathing rate. Middle panel shows the experimental normalized breathing rate percentage with respect to the averaged breathing rate in ‘pre’ epoch. Bottom panel shows the times when of each stimulation cases were applied

The peaks indicating peak inspiration were identified and used to calculate the breathing rate through the run. The breathing rates of all experimental runs in the study were collected and are represented in Fig. 4 as intensity coded raster plots. The bottom panel identifies the stimulation condition epochs corresponding to each raster. As the resting breathing rate varied between animals and runs, the raw breathing rate rasters were normalized to the average ’Pre’ epoch rate in the middle panel to enable relative comparison between runs and animals. Visually, ’LFAC only’ epoch appears identical to the ’Pre’ epoch. It shows that the LFAC waveform by itself did not change the breathing rate suggesting that it does not cause onset activation. This continues through the ‘LFAC+Stim’ epoch, although some runs such as 27 - 30 show some deviation from the ’Pre’ epoch. The lack of change during the ’LFAC+Stim’ in the raster suggests that the effects of vagal stimulation are blocked during this epoch. The ‘Stim Only’ epoch shows the effect of the HB reflex.

**Fig. 5:**
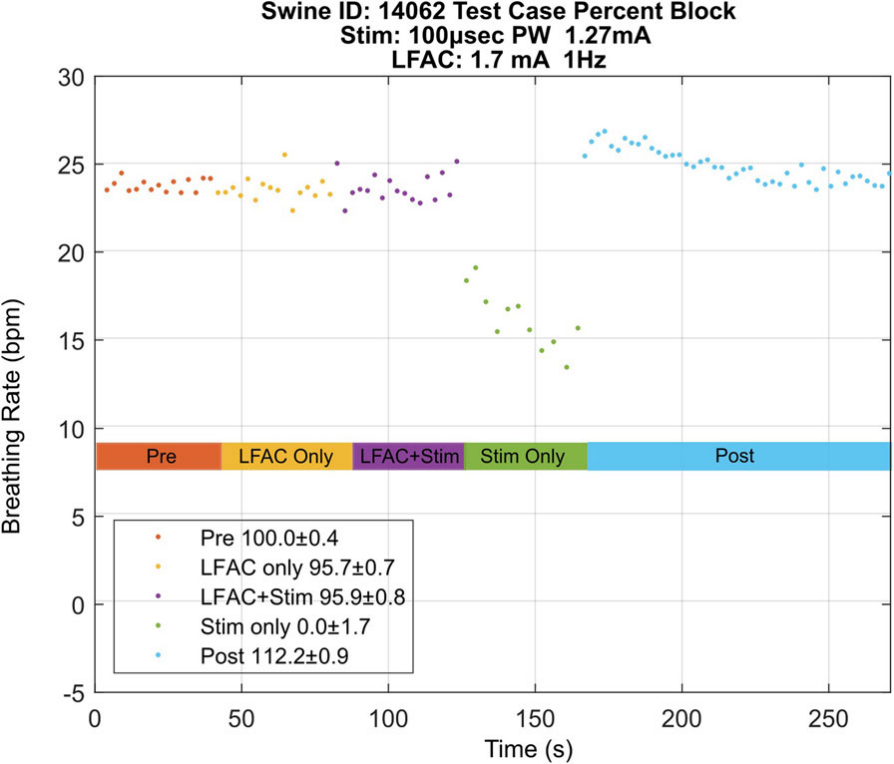
Breathing rate during each epoch of the experimental paradigm. The percent block is shown in the legend on the bottom left (± standard deviation). The ’Pre’ epoch was normalized to 100% block and the ’Stim Only’ epoch was normalized to 0% block. The overshoot seen in the first few seconds of the ‘Post’ epoch is likely due to sympathetic rebound before returning to baseline

Figure 5 shows one of the experimental runs from Fig. 4 plotted as breathing rate vs time. It is clear that application of the LFAC waveform does not change the breathing rate or cause an onset response. It also shows that during the ‘LFAC+Stim’ epoch, the breathing rate continues at a comparable rate to that of the ‘Pre’ epoch. Removal of the LFAC waveform causes an almost instantaneous drop in breathing rate due to vagal stimulation, which demonstrates the reversibility of LFACb. Discontinuing the vagal stimulus caused an overshoot likely due to sympathetic rebound before returning to baseline.

**Fig. 6:**
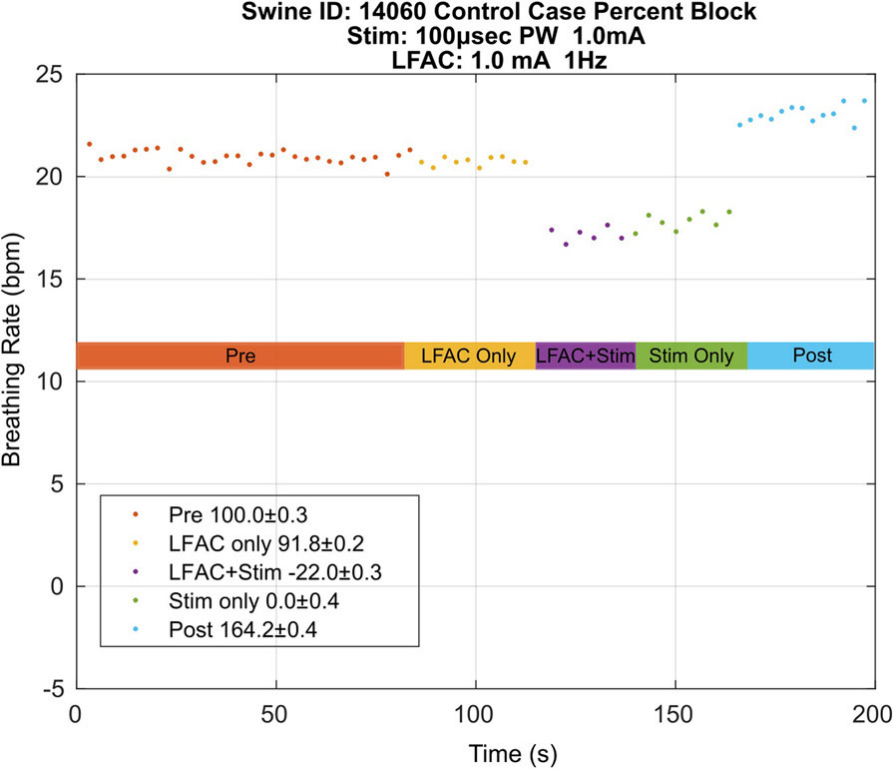
BRrate during a control case in which the electrode connections were swapped. Vagal stimulation was delivered via the CE and the LFAC waveform was presented at the RE. In this control case, the percent block (± SD) during the ’LFAC+Stim’ epoch was calculated to be -22%. A negative percent block indicates that the breathing rate during ’LFAC+Stim’ was lower than the breathing rate of the ’Stim Only’ epoch

A possible explanation for the effect of block observed could be that block is effected due to an interaction between electrodes or stimulation train and LFAC waveform. Therefore, a control case, Fig. 6, was introduced in which vagal stimulation was delivered through the RE and the LFAC waveform was presented through the CE. As in the test case, there is no onset response associated with the application of the LFAC waveform. Conversely, there is a decrease in BRrate during ‘LFAC+Stim’ indicating that the HB reflex was not blocked. Additionally, the BRrate during ‘LFAC+Stim’ and ‘Stim Only’ are approximately equal. This provides evidence that the reflex was not blocked, as was expected in the control case, indicating that the block effect is not due to an interaction between electrodes or waveforms.

Out of the 8 experiments conducted, there were 5 successful. The 3 unsuccessful experiments were excluded in the analysis. These experiments were unsuccessful due to hardware synchronization issues between the LFAC and pulse stimulation, where no evident HB reflex during vagal stimulation, or where insufficient time between runs lead to unstable respiratory chemoreceptor activity resulting in fluctuations in breathing rate (Iber et al. 1995). Table 1 summarizes the stimulation and blocking waveform parameters for the successful experiments. The average percent block achieved during ‘LFAC+Stim’ was 87.2 ±8.8%. In contrast, the control cases averaged 9.3 ±24% block during ‘LFAC+Stim’. A negative percent block indicates that the BRrate during ‘LFAC+Stim’ was lower than that of the BRrate in the ‘Stim Only’ epoch.

**Table 1:** Vagal stimulation and LFAC parameters used in the set of n=5 successful experiments. The LFAC waveform was strictly applied at 1 *Hz*. The average percent block amongst n = 5 experiment was calculated to be 87.2 ± 8.8 *%* (± SD). Two pulse widths, 1000 *μsec* and 100 *μsec*, were used for Swine ID 13525. The voltage drop across the blocking electrodes were within the Water window of the electrodes. *In one case, instrumentation issues did not allow for the retrieval of the LFAC voltage

Swine		Vagal Stimulation		LFACb Waveform		% Block
ID	PW (usec)	PA (mA)	Charge (mC)	Current (mAp)	Voltage (Vp)	Mean
21435	1000	0.22	0.22	1.00	0.20	88.23
13523	1000	1.19	1.19	1.00	0.45	94.48
13525	1000/100	0.35	0.04	0.88	0.47	86.32
14060	100	1.0	0.1	1.18	*N/A	94.20
14062	100	1.21	0.12	1.63	0.62	72.89
**Mean**		**0.8**	**0.3**	**1.1**	**0.4**	**87.2**
**SD**		**0.5**	**0.5**	**0.3**	**0.2**	**8.8**

**Fig. 7:**
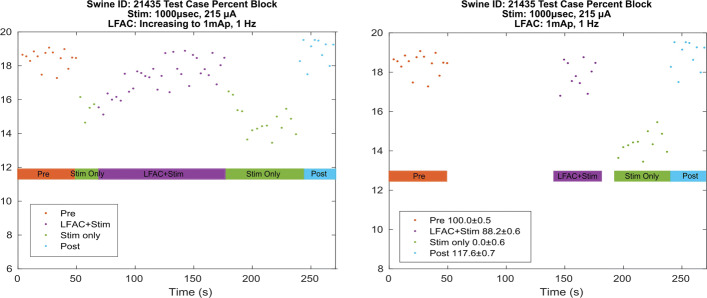
Test case of Swine ID 21435. In this unconventional case, the test sequence was applied out of order. Following a baseline reading, the HB reflex was established. The amplitude of the LFAC waveform was then slowly ramped up until block was achieved. The LFAC waveform was then discontinued which resulted in the activation of the HB reflex almost instantly. The left panel shows the unconventional test sequence order. To quantify the percent block (± SD), 4 epochs were isolated, shown on the right panel: Pre, LFAC+Stim, Stim Only, and Post. The ’LFAC+Stim’ epoch had a max amplitude of 1*mA*_*p*_ applied at 1 *Hz*, thus the window of ’LFAC+Stim’ was determined based on the location of the max amplitude

In the test case of Swine ID 21435 (Fig. 7), the test sequence was applied out of order. Following the ’Pre’ epoch, the stimulus was applied to elicit the HB reflex. After successful activation of the HB reflex, the LFAC waveform was slowly ramped up until block was achieved. Block was achieved when the waveform reached an amplitude of approximately 1mA _*p*_. Upon removal of the LFAC waveform, the HB reflex was activated again. Removal of the vagal stimulation resulted in the return to baseline respiration. In this case, the LFAC waveform was able to block an already activated reflex, which is more difficult to achieve than blocking the HB from being activated. In other words, LFAC was able to block already recruited CNAPs. Tuning of the amplitude used for demonstrating LFAC block was complicated by the subtlety of the HB reflex induced changes in breathing rate and the relatively long time it took for the change to develop. These made it difficult to see the effects of the stimulation during the course of the experiment, and were visualized clearly only during post-hoc analysis of the data. During the tuning of the block amplitude, an amplitude was chosen where clear reversal of the reflex was apparent, or the response at “Maximum” amplitude before distortion of the sinusoidal waveform.

**Fig. 8:**
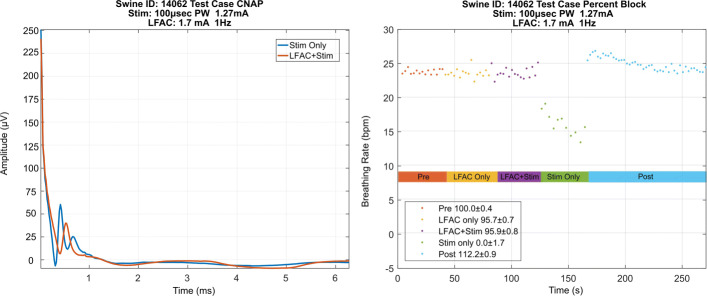
ENG recordings during an LFAC test case. In the ’Stim Only’ epoch (left panel) there are 2 clear CNAPs. The LFAC waveform during ’LFAC+Stim’ slow these CNAPs down and cause a decrease in amplitude. The right panel shows the breathing rate and percent block (± SD) during ’Stim Only’ and ’LFAC+Stim’

**Fig. 9:**
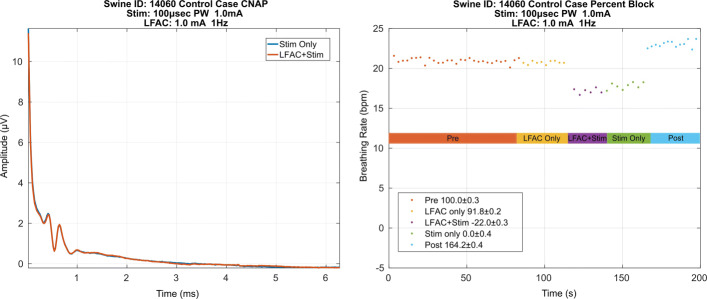
ENG recordings during an LFAC control experiment. During the ’Stim Only’ epoch (left panel) there are 2 clear CNAPs. Conversely to what occurs in the test case, the LFAC waveform in the ’LFAC+Stim’ epoch does not affect the 2 CNAPs that are visible. The CNAPs visible in the ’LFAC+Stim’ epoch are unaffected and superimposed on those seen during the ’Stim Only’ epoch, which supports the hypothesis that LFAC block does not occur due to an electrode or waveform interaction. The right panel shows the breathing rate and percent block (± SD) during ’Stim Only’ and ’LFAC+Stim’ epochs in the control case

Furthermore, the neural activity was monitored and the extraction of CNAPs was made possible due to the frequency characteristic of the LFAC waveform being outside the bandwidth of the neural activity, which is typically about 6-10 kHz (Yoshida and Struijk 2004). Figure 8 displays the effect of the LFAC waveform on the two CNAPs shown during ’LFAC+Stim’ epoch as oppose to ‘Stim Only’ epoch. It is clearly showing that there is a remarkable slowing effect and an amplitude reduction imparted by the LFAC waveform. To calculate the CNAPs conduction velocities, the distance between CE and recording electrode was measured contact-to-contact to be ∼20.86 mm during the test case and ∼10.65 mm during the control case. The conduction velocities for the 1^st^ peak and 2^nd^ peak in the ‘Stim Only’ epoch are approximately 47 and 29 m/s, respectively. However, during ‘LFAC+Stim’ the conduction velocities decrease by approximately 10 m/s to 35.7 and 18.18 m/s, respectively. The control case, Fig. 9, shows the two CNAPs in the ‘Stim Only’ and ‘LFAC+Stim’ epochs superimposed on top of each other. These observations suggest that LFAC block threshold for smaller caliber or slower fibers, like the responsible for the HB reflex (McAllen et al. 2018; Chang et al. 2015; Chang et al. 2020), is lower than that for larger fibers. It appears to block in a size wise fashion as the LFAC amplitude is increased. While CNAPs were observed on the oscilloscope during n = 5 of the experiments, neural recordings were only measured in n = 2. However, the effects shown above were consistent and repeatable throughout all experiments.

## Discussion

The aim of this study was to demonstrate the feasibility of the LFAC waveform to induce nerve conduction block in large nerve bundles. In previous work, LFAC was applied to the cervical vagus nerve of the rat where block was demonstrated against VNS induced bradycardia as a biomarker (Mintch et al. 2019). We had hypothesized that LFACb would not translate to larger diameter nerve bundles. However, in the present work, we show that block was achieved in significantly larger diameter nerve bundles than those of the rat’s cervical vagus nerve and was successful in blocking a reflex mediated by vagal afferents.

The results show that the LFAC waveform at 1 Hz achieved >85% block at average current levels of 1.1 mA_p_. In comparison, kHFACb is generally achieved at currents between 1 - 10 mA_pp_ (0.5 - 5 mA_p_) (Kilgore and Bhadra 2014; Franke et al. 2014; Kilgore and Bhadra 2004). These current levels are higher than those required for LFAC, and might contain non-monotonic threshold increases as recently found in (Peña et al. 2021). The reversibility and the absence of onset activation were verified in all test cases. The removal of the LFAC waveform causes an instantaneous drop in breathing rate due to vagal stimulation as seen in the ‘Stim Only’ epochs in Figs. 5 and 8. Furthermore, the carryover blocking effect associated with kHFACb (Franke et al. 2014; Pelot and Grill 2020; Bhadra et al. 2018; Bhadra and Kilgore 2018) was not observed, suggesting that LFAC block locally affects the peripheral nerve fibers within the cuff electrode delivering LFAC waveform without influencing central circuitry and gains. It showed no apparent carryover effect and immediately reversed upon withdrawal of presentation. The biomarker results correlated to the CNAP conduction velocity changes, which indicates that the neurological changes, slow velocity and attenuation, are due to the LFAC waveform application. The CNAP also verified that there was no onset response or activation as shown during ‘LFAC Only’ and ‘LFAC+Stim’ epochs. The absence of onset activation, a characteristic feature of kHFACb, is a key feature of the LFACb.

We used changes in breathing rate as a physiological index of vagal afferent activation. Although multiple subtypes of vagus afferent nerves can influence breathing rate (Carr and Undem 2003), the dominant response under anesthesia appears to be the HB reflex (Bouverot et al. 1970). The reduction in breathing rate we observed following activation of the vagus, and the subsequent inhibition of these changes we observed with LFAC are consistent with activation of the HB reflex. However as we did not identify the particular vagal afferent subtypes that evoked the changes we observed, it is possible that vagal reflexes other than the HB reflex were activated and therefore contributed to the changes in rate we observed. Reflexes related to activation of the glottis is unlikely since the animals were intubated, which prevents closure of the airway. Regardless of the exact vagal afferent subtypes that were activated in our experiments it is clear that LFAC was able to inhibit the observed vagal-mediated changes in respiratory rate. ECG and BP signals were used for vital signs monitoring. No changes were observed nor noted during the course of any of the experiments. These observations are in agreement with directional VNS drop induced in breathing rates without heart rate changes (Ahmed et al. 2020) achieved by the caudal nerve crush and purely rostral activation of the vagus nerve.

The CNAPs findings in Fig. 8 can be interpreted in two ways: First, during the ‘LFAC+Stim’ epoch, the first CNAP peak is attenuated and the peaks following are inverted. Second, the first peak shown is not only slowed down, but also attenuated and the second peak is also slowed down and significantly attenuated to the point of almost complete annihilation. The latter interpretation is consistent with ex-vivo and in-silico work (Horn et al. 2019), where that the LFAC waveform imposes a smearing or slowing effect on the CNAPs before block. Giving the two distinct conduction velocities of 47 and 29 *m*/*s*, complete block of the second peak resulted in the breathing rate to remain at a rate comparable to that in the ‘Pre’ epoch. Thus, these findings suggest that slower fibers were more likely the mediators of the HB reflex. Therefore, these CNAPs findings show a smearing effect prior to block and manifest a preferential order of block.

In this experiment, the left vagus nerve was crushed to eliminate cardiac reflexes while in a previous study the left vagus nerve of a rat, the nerve was crushed to eliminate cranial effects (Mintch et al. 2019). The nerve was crushed and we did not noticed any change on the heart rate. Looking at BP also did not show any changes which would normally correlate to HR changes. Mainly the ECG data were for monitoring. Crushing the nerve will not be an option in clinical applications, and standard nerve stimulation causes activation in both afferent and efferent fibers. Thus, unidirectional activation would be possible to achieve with the use of a second electrode for the delivery of LFAC to block unwanted activation.

The currents used in the LFAC conditioning waveform in this study was found to be well within the Water window of the PEDOT coated electrodes (-0.9 to 0.6 V versus Ag/AgCl) (Cogan 2008). The range is typically defined as the half-cell potential where electrodes operate linearly without reaching electrolysis of water. Unlike DC block, the LFAC waveform reverses the Faradaic reactions that occur at the electrode interface every half cycle. If left unreversed, these reactions can lead to accumulation of charge and reaction products that can damage the nerve. Nerve damaged by these reaction products usually presents as a discoloration of the tissue, and irreversible loss of nerve function. In our study, the removal of the blocking cuffs showed no observed damage nor discoloration. Furthermore, the blocking LFAC waveform was applied continuously during two epochs with no interruption to the nerve function. Upon discontinuation of the LFAC waveform, the breathing rate dropped due to the vagal stimulation for a short period of time indicating the absence of any loss of function of the nerve. Even though each experiments lasted for more than 5 hours, the animals maintained normal respiratory rate between trials. These observations suggest that the use of the LFAC waveform was not damaging to the nerve tissue.

Although these findings are encouraging, the low frequencies used require neural interfaces with exceptionally low frequency impedances with the capability of safely delivering reversible Faradic currents. The interface matters greatly and we speculate that we would likely not have been able to achieve block if standard electrode materials such as stainless steel or even platinum was used. The exceptional low frequency performance and durability of Amplicoat^®^ PEDOT coated electrodes greatly enhanced the current ranges we could apply in our study. Monitoring the two point voltage waveform to verify that the electrodes were within the linear region of operation was critically important to prevent damage to the nerve. The voltage ranges and the linearity of the waveform were confirmed with in-vitro cyclic voltammetry measurements to an effective estimate of being within the Water window limits of the electrode. While developing the preparation in pilot work, we observed that if charge was allowed to accumulate, a DC offset potential developed, the voltage drop across the LFAC electrodes became non-linear to the input current, and the sinusoid measured in the two point voltage monitor began distorting. If not immediately reversed, the nerve function irreversibly changed and the biomarker response to vagal stimulation stopped. The presence of a response to vagal stimulation in the phase without the conditioning LFAC block waveform in our study was used in part, as a control measure that the nerve was not functionally altered by the application of a balanced LFAC waveform. Thus, care must be taken when applying LFAC. We applied LFAC on the nerve over a period of 4 - 6 hours without altering nerve function, but longer term effects are unclear. Unlike pulse stimulation, which relies heavily on the non-Faradaic mechanisms such as discharging the double layer capacitance to accomplish a large portion of the charge transfer, LFAC relies on reversed Faradaic currents where the electrode potential is monitored to discount the possibility of accumulated charges. The charge per phase safety developed for pulse stimulation are likely mechanistically not directly translatable. Thus, work to assess the effects of longer term exposure to LFAC, the development of automatic methods to assure discharging of residual charges, and the development of safety metrics are future directions that need to be addressed.

## Conclusion

Our findings demonstrated the effectiveness of the LFAC waveform to achieve successful nerve conduction block of HB reflex mediated by the vagus nerve. These findings showcase LFAC as a potential alternative or complementary method to other electrical blocking techniques in clinical applications due to the features such as absence of onset response, charge-balanced waveform, and low threshold characteristics. With development and refinement, the technique could become an attractive means to achieve reversible, subtractive neural modulation.

## Data Availability

The datasets used and/or analysed during the current study are available from the corresponding author on reasonable request.

## Abbreviations

LFAC: Low Frequency Alternating Current
CNAP: Compound Nerve Action Potential
kHFACb: kilohertz Frequency Alternating Current Block
DC: Direct Current
BP: Blood Pressure
ECG: electrocardiograph
RE: Rostral Electrode
CE: Caudal Electrode
PEDOT: poly-ethylenedioxythiophene
HB: Hering-Breuer
BRrate: Breathing Rate
FM: Frequency Modulation
IACUC: Institutional Animal Care and Use Committee
PW: Pulse Width
PA: Pulse Amplitude
